# When crisis hits: Bike-Sharing platforms amid the Covid-19 pandemic

**DOI:** 10.1371/journal.pone.0283603

**Published:** 2023-04-07

**Authors:** Ecem Basak, Ramah Al Balawi, Sorouralsadat Fatemi, Ali Tafti

**Affiliations:** 1 Zicklin School of Business, Baruch College, City University of New York, New York, New York, United States of America; 2 College of Business Administration, University of Illinois at Chicago, Chicago, Illinois, United States of America; Qatar University, QATAR

## Abstract

In this work, we examine the changes in demand for bike-sharing platforms with the onset of the Covid-19 pandemic. Using the fixed-effects regression formulation of difference-in-differences, we evaluate how the demand for bike-sharing platforms changed after the first cases of Covid were discovered and after the first executive orders were implemented. Accounting for weather conditions, socio-economic characteristics, time trends, and fixed effects across cities, our findings indicate that there is an increase in daily bike-sharing trips by 22% on average after the first Covid-19 case diagnosis, and a decrease of 30% after the first executive order implementation in each municipality, using the data up to August 2020. Moreover, we observe a 22% increase in weekday-specific trip frequency after the first Covid-19 case diagnosis and a 28% decrease in weekend-specific trip frequency after the first executive order implementation. Finally, we find that there is an increase in the frequency of trips on bike-sharing platforms in more bike-friendly, transit-friendly, and pedestrian-friendly cities upon both the first Covid-19 case diagnosis and the first executive order implementation.

## Introduction

The Covid-19 pandemic has significantly affected our societal and economic structures. Mandated lockdowns and voluntary precautions, which are taken to reduce the spread of the virus, have affected the demand for all modes of transportation, including public transport in cities. For example, Aloi et al. [1] indicate a fall of 76% in overall human mobility and a 93% decrease in public transport usage in Santander, Spain. Using aggregated mobility data from mobile phones in numerous urban areas in the U.S., Kishore et al. [2] show a surge in travel out of the cities immediately preceding the stay-at-home advisory. Another study points out a significant reduction in traffic volumes of 30% to 50% for select highways in California compared to prior shelter-in-place orders [3].

Individuals have changed their transportation patterns as personal travel decisions affect the spread of Covid-19 [4]. In response to the social distancing order, people have been less inclined to board packed buses and trains where social distancing is impossible. Accordingly, individuals reevaluate their transportation options in the face of the Covid-19 pandemic and shift to more isolated modes such as biking or walking.

With the Covid-19 pandemic, we witness an increasing awareness of bicycles as an alternative means of transport, as many people either avoid using mass transit or encounter reduced mass transit services. In the U.S., sales of bicycles and related equipment also almost doubled in March 2020 compared with the same period in 2019 [5]. In times of crisis, bicycles can provide resilience in transport systems, satisfying our mobility needs when mass transit systems are inaccessible. For instance, during the national public transit strike in France in December 2019, Parisians adapted and learned that bikes are dependable and credible modes of transport. The bike-sharing system in Paris, Vélib, gained popularity during the strike [6]. Other examples are the 2005 New York City transit worker strike and Hurricane Sandy in 2012, which severely disrupted New York’s subway system. These events led to an increase in bicycle ridership in the city of New York by about 20% [7]. Bicycle sales surged in Japan after earthquakes struck that country in 2011 [8].

With the surge in demand for bikes, the popularity of bike-sharing platforms has also increased in March compared to the same period in the year before. They have become a viable transport alternative that reduces the risk of contracting or spreading the virus and relieves the fear of overcrowding [9, 10]. Compared to other means of transportation systems such as buses or trains, bicycling is an open-air activity and helps to avoid close contact with other travelers. Therefore, people have a more positive attitude toward bike-sharing for traveling amidst the pandemic [11]. For instance, Citi Bike in New York City announced a 67% increase in demand between March 1, 2020, and March 11, 2020, compared with the same period in 2019. Divvy in Chicago has also reported that the number of trips doubled in the same period [12]. A report from Foursquare and Apptopia shows that bike-share mobile application installations in May and June of 2020 were up 15.6% and 23.3%, respectively, compared to the prior year [13]. A recent study by Li et al. [14] analyzed the demand for the bike-sharing platform in London over the period from January 2019 to June 2020. They found that the number of bike-sharing trips in London decreased after the lockdown; however, it was followed by an increase in demand over time. Heydari et al. [15] investigated the impact of the Covid-19 pandemic on the London bike-sharing platform over the period from March 2020 to December 2020. They initially observed a reduction in bike trips between March and April 2020; however, demand increased in May and June 2020.

Moreover, Bouhouras et al. [16] found that the demand for bike-sharing platforms in Greek cities such as Igoumenitsa, Chania, and Rhodes increased in a short period of time before the lockdown period and peaked during the lockdown. Wang and Noland [17] examined the impact of Covid-19 on both bike-sharing and subway usage. They found that both subway ridership and bike-sharing usage plummeted at the beginning; however, bike-sharing usage has almost returned to normal, whereas subway ridership has remained substantially below pre-pandemic levels. Other recent studies [18, 19] also revealed that bike-sharing platform usage in many cities has reached or surpassed pre-pandemic levels [20].

It is argued that bike-sharing demand has plummeted because of lockdown waves, even though it shows higher resiliency and lower drop than subway systems [10]. Following the stay-at-home advisories, many companies began to allow their employees to work from home, resulting in a significant reduction in travel within cities. Studies suggest that the demand for bike-sharing platforms has also decreased due to increased levels of remote working and stay-at-home advisories, but not as much as other means of transportation. A study conducted in Budapest, Hungary, shows that there has been an 80% decrease in public transport demand and only a 2% reduction in the use of bike-sharing platforms during the pandemic [21]. Another study that used ridership data from New York in 2020 showed that bike-sharing trips have decreased by less than 71%, whereas subway trips have decreased by 90% compared to February and March of 2019 [10].

Another study by Li et al. [22] examined the changes in demand for micro-mobility services such as bike-sharing platforms in Zurich, Switzerland, before and during the lockdown period. Their spatial and temporal analysis results showed a decrease in the number of trips during the lockdown period. Their study also revealed that leisure- and shopping-related micro-mobility trips decreased while grocery-related trips increased. Apple mobility data shows that, by the end of May 2020, there was a decrease in all modes of transportation including driving, walking, public transit, and bike-sharing; however, the reduction in public transit ridership was down much more than bike-share usage [23]. According to a press release by the Bureau of Transportation Statistics, ridership on eight of the largest docked bike-share systems in the U.S. declined by 44% from March through May 2020, compared to the same period in 2019 [24]. This could be explained by the fact that people have been traveling less due to stay-at-home advisories and limited business operations, and this might be affecting the demand for bike-sharing platforms like other transportation modes.

In this paper, we examine how demand for bike-sharing platform usage changed immediately following the first Covid-19 case and the first executive order in the U.S., using the data from January 2019 to July 2020. We use a fixed-effects econometric formulation of the difference-in-differences (DID) estimation framework, which exploits a natural experiment to examine how bike-sharing trips have changed with the introduction of the first Covid-19 case and the first executive order. The DID estimation method is a suitable technique in our context, where randomization on the city level is not possible. DID requires panel data, which is part of the fixed-effects strategy, to capture the differences in post-treatment periods across the treatment and control groups [25]. One benefit of the DID model is that it allows us to avoid “the endogeneity problems that typically arise when making comparisons between heterogeneous individuals” [26].

We also consider how the frequency of bike-sharing platform use can be different on weekdays compared to weekends due to the changing travel patterns during the pandemic. In general, weekday travel is primarily made up of commuting to and from work, whereas weekend travel behavior is motivated by recreational activities. Differences in activity types can lead to different travel patterns that can be hypothesized on weekends and holidays, compared to weekdays [22, 27, 28]. For instance, Agarwal [27] suggests that there is a decrease in vehicle trips on weekends compared to weekdays at the household level. Kim et al. [28] find different weekend and weekday bike-sharing patterns. Their results point out that there is an increase in bike-sharing traffic volume on the weekends at the stations near parks and schools, which can be due to the rise in leisure and school activities on the weekends. In contrast, residential areas and subway stations are found to have less bike-sharing traffic volume on the weekends than on weekdays. Li et al. [22] find that there was a decrease in the number of micro-mobility service trips on weekdays during the lockdown period in Zurich. In contrast, there are only slight changes on weekends compared to before the lockdown period.

In addition, we investigate what factors strengthen or weaken the impact of the pandemic on the frequency of bike-sharing use. Transportation infrastructure, land use, and neighborhood attributes contribute to individuals’ preference for bike-sharing [29]. Several studies examine the effects of the built environment, cycling facilities, transit proximity, and transportation facility features on bike-sharing frequency [30–32]. The findings are consistent: More bicycle facilities and more excellent transit proximity lead to greater use of bike-sharing. Recent studies [33, 34] also find that better biking infrastructure is linked to higher bike-sharing demand during the Covid-19 pandemic. For instance, according to Bergantino et al. [33], safer cycling conditions and the creation of dedicated infrastructures encourage individuals to use bike-sharing platforms during the pandemic. Therefore, we also test the heterogeneous effects depending on such factors as the city’s bike-friendliness, transit-friendliness, and pedestrian-friendliness.

The remainder of the paper is organized as follows. The next section introduces the data collection, variable definitions, and research method. Section 3 presents the main results. Section 4 shows the heterogeneous effects of bike-friendliness, transit-friendliness, and pedestrian-friendliness. Finally, Section 5 concludes with a discussion of the findings.

## Materials and methods

### Data and variables

We collected data from multiple sources. First, we collected the historical daily trip data available to the public from bike-sharing programs in Austin, Boston, Chicago, Columbus, Minneapolis, New York, Philadelphia, Pittsburgh, Portland, San Francisco, and Washington, D.C. The daily trip data includes trip duration, start time, end time, starting station, ending station, and subscription type (*i*.*e*., *member*, *single rider*). Based on this data, we compute our dependent variable, the daily trip frequency of bike-sharing platform trips (*TripFrequency*_*ij*_*)*. It is calculated as the total daily trips of bike-sharing platform *i* at time *j*. During the construction of this variable, we excluded the bike-sharing platform trips with a duration of two minutes or less as there might be an issue while renting the bike (*i*.*e*., *the bike is in a bad condition*). We also excluded the bike-sharing platform trips of forty-five minutes or more, as these trips are more likely to represent leisure and recreational trips. A single ride for subscribers of these services includes forty-five minutes of ride time.

Second, we collected data from various online sources to construct our treatment measures, *FirstCase*_*ij*_ and *FirstExecutiveOrder*_*ij*_. Our first treatment variable is the first Covid-19 case diagnosis (*FirstCase*_*ij*_), which is coded as 1, indicating that the first Covid-19 case is identified in city *i* as of day *j’* such that *j* > = *j’*. The data on Covid-19 cases comes from The New York Times [35], which is based on reports from state and local health agencies. Our second treatment variable is *FirstExecutiveOrder*_*ij*_, which is coded as 1, indicating that an executive order is issued in city *i* as of day *j’* such that *j* > = *j’*. Specifically, we refer to the first executive action taken by the state governments against the Covid-19 pandemic, which is the stay-at-home advisories announced by the state governments. While stay-at-home advisories were lifted before August 2020, restrictions continued in most cities in various forms. For instance, Minnesota’s stay-at-home advisory expired on May 18, 2020; however, it was replaced with a "stay safe Minnesota" order. Moreover, the state extended the state of emergency by another 30 days. Therefore, as the restrictions were still in effect, cities did not fully reopen before August 2020. Consequently, we used the entire period after the first executive order implementation. Table 1 lists the start date for each of our treatment variables in each city in our study.

**Table 1:** Treatment start dates.

City	First Covid-19 case diagnosis	First executive order
Austin, TX	03/13/2020	03/24/2020
Boston, MA	02/01/2020	03/24/2020
Chicago, IL	01/24/2020	03/21/2020
Columbus, OH	03/14/2020	03/23/2020
Minneapolis, MN	03/12/2020	03/27/2020
New York, NY	03/02/2020	03/22/2020
Philadelphia, PA	03/09/2020	03/23/2020
Pittsburgh, PA	03/14/2020	03/23/2020
Portland, OR	03/10/2020	03/23/2020
San Francisco, CA	03/05/2020	03/17/2020
Washington D.C.	03/08/2020	04/01/2020

To construct the control variables, we collected weather data from the National Oceanic and Atmospheric Administration (NOAA). Control variables are included in the model as pre-treatment variables, as weather conditions can have different impacts on the demand for bike-sharing trips. Evidence from empirical studies [36–39] indicates that favorable weather conditions, such as higher temperatures, increase bike-sharing platform usage. In contrast, unfavorable conditions, such as precipitation and strong winds, will decrease such use. For example, Gebhart and Noland [36] suggest that cold temperatures, rain, and high humidity levels are likely to reduce the demand for bike-sharing platform trips in Washington, DC. In contrast, high temperatures are linked to an increased number of such trips. Similarly, the findings of Morton [37] point out that higher temperatures are associated with higher demand rates, whereas heavy precipitation, high wind speed, and relative humidity are negatively associated with the demand for the London bike-sharing system. Consistent with previous studies, An et al. [39] find out that there is a higher demand for the CitiBike bike-sharing platform in NYC in good weather, which is characterized by favorable temperature conditions, lack of winds, humidity, and rain. On the other hand, El-Assi et al. [31] show that weather conditions such as precipitation and high humidity decrease the demand for the Toronto bike-sharing system. Based on the background evidence, first, we control for the following weather-related variables: 1) *Temperature*_*ij*_, a measure of the average temperature for day *j* in city *i* in Fahrenheit (°F); 2) *Wind*_*ij*_, a measure of the average wind speed for day *j* in city *i* in knots; 3) *Snow*_*ij*_, a measure of snow depth for day *j* in city *i* in inches; 4) *Rain*_*ij*_, a measure of total precipitation for day *j* in city *i* in inches; and 5) *Humidity*_*ij*_, a measure of the average dew point for day *j* in city *i* in Fahrenheit (°F).

Furthermore, we collected data on the socio-economic characteristics of the cities from the U.S. Census Bureau. We include the population (*Population*_*ij*_), median income (*Income*_*ij*_), the number of the elderly population (*Elderly*_*ij*_), the number of houses with two cars (*Vehicle*_*ij*_), and the number of people commuting to work with bikes (*Commute*_*ij*_).

When combined, we end up with a panel data set that comprises eleven cities spanning from January 2019 through July 2020. To make the interpretation of the socio-economic characteristics easier, we include the log-transformed values in our analyses. Following prior literature, we keep the weather-related variables in their original form [36, 37, 39]. Tables 2 and 3 present the summary statistics and the correlation of the critical variables, respectively. We use the log-transformed values of the socio-economic characteristic to interpret linear regression results.

**Table 2:** Descriptive statistics.

Variable	Obs.	Mean	Std. Dev.	Min	Max
FirstCase	6,052	0.26	0.44	0.00	1.00
FirstExecutiveOrder	6,052	0.23	0.42	0.00	1.00
ln(TripFrequency)	6,052	7.27	2.06	0.00	11.45
ln(Population)	6,052	15.15	0.75	14.25	16.75
ln(Elderly)	6,052	13.22	0.81	12.05	14.92
ln(Income)	6,052	11.28	0.17	11.01	11.62
ln(Vehicle)	6,052	13.14	0.60	12.39	14.32
ln(Commute)	6,052	9.75	0.96	7.81	11.07
Temperature	6,052	56.89	16.49	-13.50	91.00
Rain	6,052	0.12	0.30	0.00	4.42
Snow	6,052	0.08	0.51	0.00	9.10
Wind	6,052	7.23	3.45	0.50	24.00
Humidity	6,052	44.19	16.89	-24.60	77.90

**Table 3:** Correlation.

	**Variable**	**1**	**2**	**3**	**4**	**5**	**6**	**7**	**8**	**9**	**10**	**11**	**12**	**13**
**1**	FirstCase	1.00												
**2**	FirstExecutiveOrder	0.90	1.00											
**3**	ln(TripFrequency)	-0.07	-0.08	1.00										
**4**	ln(Population)	0.03	0.00	0.87	1.00									
**5**	ln(Elderly)	0.05	0.02	0.85	0.95	1.00								
**6**	ln(Income)	0.12	0.10	0.51	0.27	0.21	1.00							
**7**	ln(Vehicle)	0.03	0.00	0.85	0.96	0.95	0.21	1.00						
**8**	ln(Commute)	0.02	-0.01	0.86	0.78	0.75	0.66	0.74	1.00					
**9**	Temperature	0.16	0.24	0.13	-0.09	-0.12	0.08	-0.09	-0.04	1.00				
**10**	Rain	0.00	0.00	-0.02	0.02	0.02	-0.05	0.03	-0.03	0.02	1.00			
**11**	Snow	-0.05	-0.07	-0.05	0.07	0.07	-0.07	0.09	0.00	-0.27	0.01	1.00		
**12**	Wind	0.05	0.03	0.08	0.11	0.13	0.18	0.12	0.14	-0.23	0.11	0.06	1.00	
**13**	Humidity	0.12	0.20	0.08	-0.12	-0.14	0.05	-0.13	-0.06	0.93	0.12	-0.22	-0.24	1.00

### Model-free evidence

**Fig 1:**
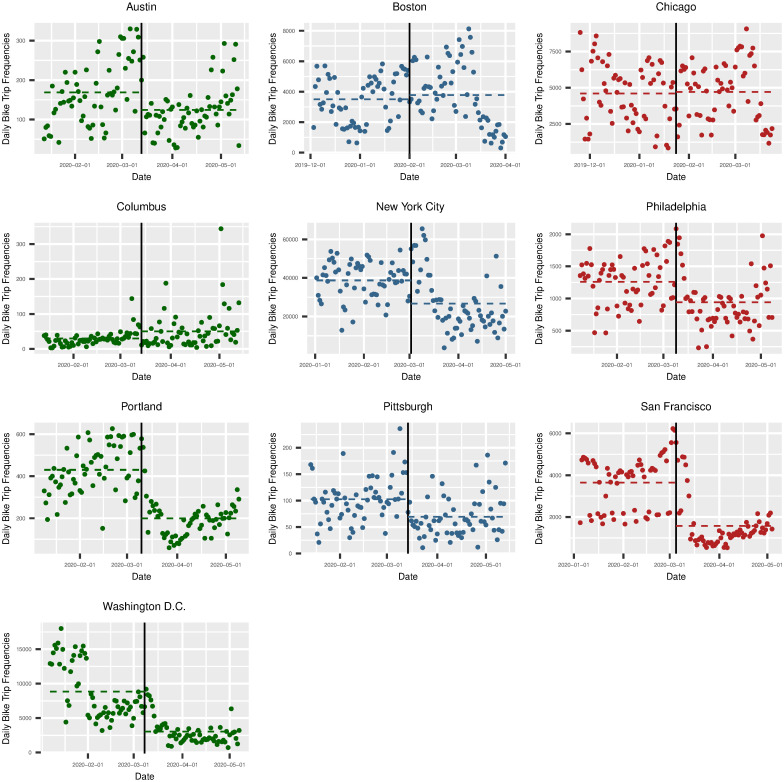
Frequency of bike-sharing platform trips over time, before and after the first Covid-19 case.

**Fig 2:**
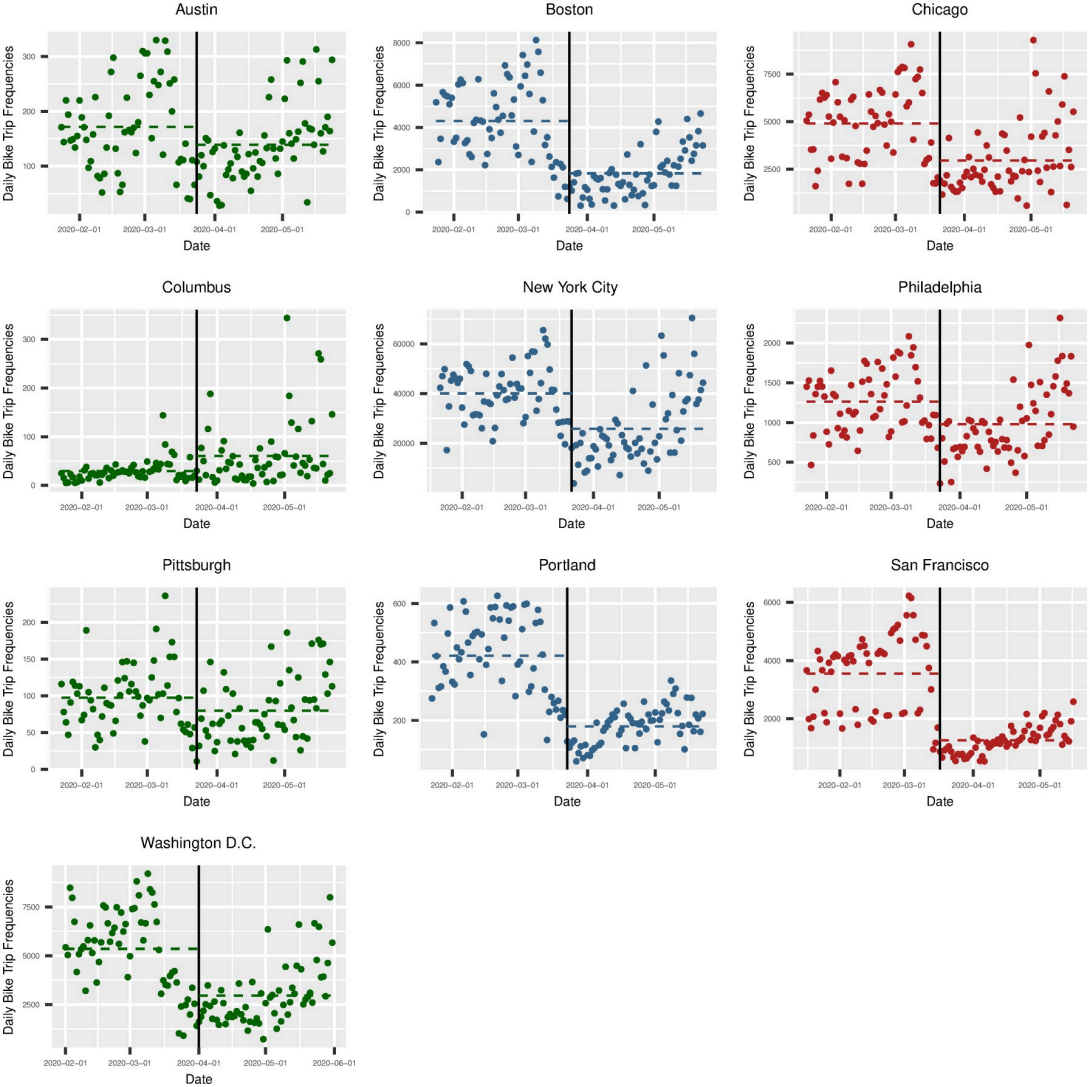
Frequency of bike-sharing platform trips over time, before and after the first executive order implementation.

Before we introduce our model specification, we present visual model-free evidence of the role of the first Covid-19 case diagnosis and the first executive order implementation on the use of the bike-sharing platforms in Figs 1 and 2. It is worth noting that Figs 1 and 2 do not account for time-fixed effects. In Fig 1, we plot the daily trip frequency 60 days before and after the first reported Covid-19 case in each of the eleven cities (excluding Minneapolis, MN, as its bike-sharing systems do not report daily trip data between December and March). The solid vertical line represents the first Covid-19 diagnosis. The dashed horizontal lines represent the average daily bike-sharing trip frequency before and after the first Covid-19 diagnosis. In Fig 1, we observe that the daily bike trip frequency decreases in most cities following their first reported Covid-19 case, except for Boston and Chicago, which had cold winters and were beginning to warm up in the ensuing weeks. In Fig 2, we plot the daily bike trip frequency 60 days before and after the first executive order implementation across the cities. We observe a similar trend in Fig 2. Across all cities, the daily trip frequency declined in the days immediately after the first executive orders were implemented.

Similar to the plots in Figs 1 and 2, we plot the difference in the bike-sharing trip frequency before and after the first Covid-19 case diagnosis and the first executive order implementation by weekday and weekend (*see Fig A1 and A2 in* S1 Appendix *for more details*). In Fig A1 in S1 Appendix, we show the *weekday* bike trip frequencies 60 days before and after the first Covid-19 case reported in each city. We notice a decrease in the trip frequency on weekdays following the first reported Covid-19 case in most cities, with a few exceptions in which we see a close average daily trip frequency after the first Covid-19 case was reported, such as in Boston. In Fig A2 in S1 Appendix, we show the *weekend* bike trip frequencies 60 days before and after the first Covid-19 case that was reported. When we look at the changes in the weekend trip frequency, we see opposing results suggesting an increase in the daily trip frequency in some cities, such as Philadelphia and Pittsburgh.

Moreover, we also notice a similar trend in the daily weekday and weekend trip frequency after the first executive order implementation across the cities in this study (*see Figs A3 and A4 in* S1 Appendix). Finally, Fig A5 in S1 Appendix shows the bike-sharing seasonal trend of February-June 2019 (pre-covid) compared to February-June 2020 (post-covid) for each city in this study. Relative to the patterns observed in 2019, we see a short-term decrease in bike-sharing trip frequency following the pandemic’s start (towards the end of the first quarter of 2020). These plots provide further model-free evidence of the changes in the use of the bike-sharing system due to Covid-19.

**Fig 3:**
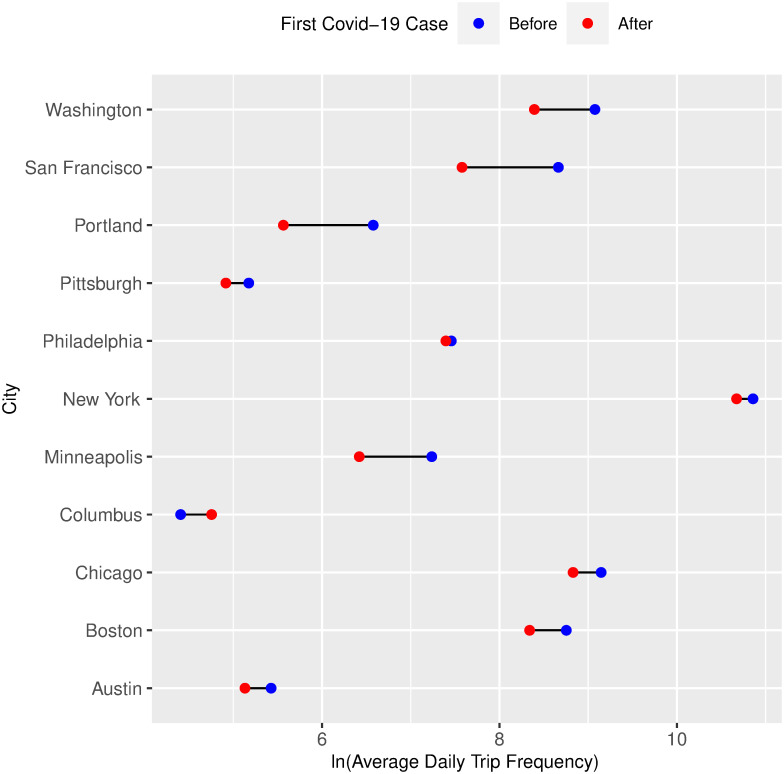
Dumbbell chart comparing the average daily bike-sharing trip frequency before and after the first Covid-19 case.

**Fig 4:**
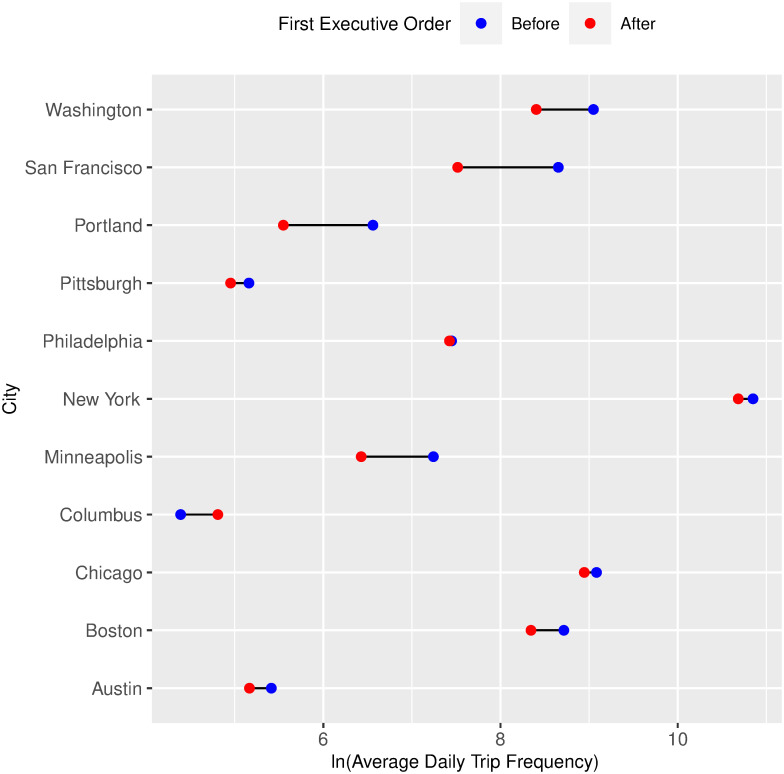
Dumbbell chart comparing the average daily bike-sharing trip frequency before and after the first executive order implementation.

Figs 3 and 4 show dumbbell charts that compare the average daily bike-sharing trip frequency before and after each of our two treatments (the first Covid-19 case diagnosis and the first executive order implementation) that occurred over the entire period of study. We use the log transformation of trip frequencies for better visualization. Generally, we see short-term decline in bike-sharing frequency after the first reported infections and the first executive order implementation within the same U.S. cities. The blue point represents the log average daily trip frequency for the period ***before*** the treatment occurred, and the red point represents the log average daily trip frequency in the period ***after*** the treatment occurred. Fig 3 shows that the frequency of average daily bike-sharing platforms decreases after the first Covid-19 case diagnosis, except for a few cities such as New York, Philadelphia, and Chicago, in which the average daily bike-sharing trips did not change significantly; and also except for Columbus, in which we see an apparent increase. Fig 4 also shows that the frequency of average daily bike-sharing trips decreases upon the first executive order implementation, again with the aforementioned exceptions. However, it is essential to note that these plots do not consider the cities’ weather conditions and socio-economic characteristics. Therefore, in the following subsection, we propose a statistical model to evaluate how bike-sharing frequency changed following the Covid-19 pandemic, accounting for weather conditions and socio-economic characteristics.

### Model specification

The model-free evidence shows that the frequency of trips on bike-sharing platforms generally decreased in U.S. cities following the first Covid-19 cases and the first executive order implementation. However, as mentioned earlier, model-free evidence does not account for many factors that could influence the trip frequency for bike-sharing and the spread of the virus. Therefore, before incorporating such factors into our statistical analysis, we need to know the pandemic’s actual effect on bike-sharing trip frequency. However, we may observe a decrease in the trip frequency in some cities and an increase in others.

Thus, we use a fixed-effects econometric formulation of the DID estimation framework to examine how the Covid-19 pandemic affected the frequency of bike-sharing trips. The primary benefit of this estimation model is that we can mimic an experimental design using observational data. This method compares the differences in bike-sharing trip frequency in treated cities before and after the treatment event—the onset of the pandemic—to the differences in the untreated cities (*i*.*e*., those cities yet to report a coronavirus case or to implement a first executive order). The longitudinal nature of the data allows us to use the yet untreated observations in the data as controls for the treated observations; that is, those cities that have *yet* to have a first Covid-19 case or a Covid-related executive order. To facilitate estimation, we use the fixed-effects regression formulation of the DID model, a formulation described in [25], as follows:

$$ln{\left(TripFrequency\right)}_{ij}=\;\beta_{0}+\beta_{1}Treatment_{ij}+\;\gamma W_{ij}+\;\theta_{j}+\;\mu_{i}\;+\varepsilon_{ij},$$ (1)

where ln(*TripFrequency)*_*ij*_ is the log-transformed value of our dependent variable in city *i* during day *j*. *Treatment*_*ij*_ refers to the treatment variables in city *i* during day *j*: *FirstCase*_*ij*_ or *FirstExecutiveOrder*_*ij*_. They are applied in different cities at different times. To control for existing time-invariant differences among the heterogeneous geographical locations, i.e., cities, we included city-fixed effects, *μ*_*i*_, in our model. In addition, we included time-fixed effects, *θ*_*j*_, to control for common temporal shocks. This allows for non-linear time-varying effects in the DID model. *W*_*ij*_ is the set of control variables, which includes ln(*Population*), ln(*Income*), ln(*Elderly*), ln(*Vehicle*), ln(*Commute*), *Temperature*, *Rain*, *Snow*, *Wind*, and *Humidity*. Finally, *ε*_*ij*_ is the error term.

## Results

Table 4 reports the coefficient estimates of Eq (1) for the dependent variable ln(*TripFrequency*). As shown in Column (1), we estimate an increase in the log of bike-sharing platform trip frequency of 0.196 on average across eleven cities after the first Covid-19 case, adjusted for covariates. An economic interpretation of this result suggests an average adjusted increase in the number of daily bike-sharing trips by 22% (rounded from the following: [exp(0.196)-1]*100 = 21.65%). On the other hand, from Column (2), we observe a decrease in the log of bike-sharing platform trip frequency by 0.353 after the stay-at-home order implementation. Economically, this result suggests a reduction of 30% (rounded from the following: [exp(-0.353)-1]*100 = -29.74%) in the number of daily bike-sharing trips. We also further examine the robustness of our model to temporal trends using a relative time model (*see Fig A6 and Fig A7 in* S1 Appendix *for more details*).

**Table 4:** Bike-sharing trip frequency: Fixed-effects regression results.

	(1)	(2)
Dependent Variable	ln(TripFrequency)	ln(TripFrequency)
*Treatment variable*	*First Case*	*First Executive Order*
	*0*.196**	*-0*.353*
	*(0*.*098)*	*(0*.*205)*
ln(Population)	-1.748	-4.171
	(14.555)	(14.779)
ln(Elderly)	7.876	8.446
	(9.519)	(9.667)
ln(Income)	6.751*	7.408**
	(3.567)	(3.652)
ln(Vehicle)	7.591***	7.355***
	(2.505)	(2.502)
ln(Commute)	0.332	0.357
	(0.429)	(0.437)
Temperature	0.043***	0.044***
	(0.003)	(0.003)
Rain	0.012	0.012
	(0.022)	(0.021)
Snow	-0.172***	-0.170***
	(0.038)	(0.038)
Wind	-0.026***	-0.026***
	(0.005)	(0.005)
Humidity	-0.017***	-0.017***
	(0.003)	(0.002)
Observations	6,052	6,052
R^2^	0.284	0.283
R^2^(Adjusted)	0.206	0.205
F-statistic	197.145***	196.389***
Daily fixed effects	Yes	Yes
City fixed effects	Yes	Yes

*** p<0.01

** p<0.05

* p<0.10

Robust standard errors are given in parentheses.

Furthermore, we divide our dataset into three panels to compare weekday, weekend, and bank holiday travel behavior. We include the bank holidays (*i*.*e*., *New Year’s Day*, *Martin Luther King Jr*. *Day*, *or Independence Day*) observed by the Federal Reserve System. The results are given in Tables 5 and 6. We estimate a 22% (rounded from the following: [exp(0.197)-1]*100 = 21.77%) increase in the trip frequency on average across cities during the weekdays upon the first Covid-19 case (*see Column 1 in* Table 5), whereas our results do not suggest a statistical significance of the same effect on weekends (*see Column 2 in* Table 5) and the bank holidays (*see Column 3 in* Table 5). Table 6 shows the results when the treatment variable is *FirstExecutiveOrder* in which we observe opposing results. We estimate a statistically significant decrease in the trip frequency of 28% (rounded from the following: [exp(-0.323)-1]*100 = -27.60%) on the weekends (*see Column 2 in* Table 6), whereas we find no evidence of the same effect during the weekdays (*see Column 1 in* Table 6). However, we do not observe any effect for the bank holidays data panel as the variable in question is being omitted by the regression (*see Column 3 in* Table 6). The reason behind this is that any variables that are constant within every unit are redundant in a fixed-effects model and will be omitted from the model. Due to the launch dates of the first executive order, Memorial Day 2020 and Independence Day 2022 are treated the same for each unit. Therefore, our treatment variable becomes constant for each city and does not create any variation.

**Table 5:** Bike-sharing trip frequency: Weekday-, weekend-, and bank holiday-specific fixed-effects regression results where the treatment is the first Covid-19 case.

	(1)	(2)	(3)
Dependent Variable	ln(TripFrequency)	ln(TripFrequency)	ln(TripFrequency)
Travel Behavior	Weekday	Weekend	Bank Holiday
*Treatment variable*	*First Case*	*First Case*	*First Case*
	*0*.197**	*0*.*174*	*0*.*159*
	*(0*.*098)*	*(0*.*113)*	*(0*.*420)*
ln(Population)	-3.378	0.892	0.824
	(13.776)	(18.152)	(29.735)
ln(Elderly)	10.125	4.440	-13.653
	(9.625)	(10.328)	(17.933)
ln(Income)	6.780**	7.152	2.036
	(2.961)	(5.179)	(7.694)
ln(Vehicle)	7.441***	8.527**	-0.633
	(2.145)	(3.777)	(5.811)
ln(Commute)	0.440	0.007	1.003
	(0.392)	(0.674)	(1.025)
Temperature	0.037***	0.059***	0.061***
	(0.002)	(0.008)	(0.010)
Rain	0.006	0.019	0.141
	(0.016)	(0.039)	(0.234)
Snow	-0.158***	-0.175***	-0.199***
	(0.032)	(0.036)	(0.068)
Wind	-0.024***	-0.028***	-0.039**
	(0.005)	(0.006)	(0.016)
Humidity	-0.014***	-0.025***	-0.028***
	(0.002)	(0.003)	(0.009)
Observations	4,161	1,736	155
R^2^	0.271	0.337	0.438
R^2^(Adjusted)	0.189	0.257	0.273
F-statistic	126.577***	71.746***	8.443***
Daily fixed effects	Yes	Yes	Yes
City fixed effects	Yes	Yes	Yes

*** p<0.01

** p<0.05

* p<0.10

Robust standard errors are given in parentheses.

**Table 6:** Bike-sharing trip frequency: Weekday- and weekend-specific fixed-effects regression results where the treatment is the first executive order.

	(1)	(2)
Dependent Variable	ln(TripFrequency)	ln(TripFrequency)
Travel Behavior	Weekday	Weekend
*Treatment variable*	*First Executive Order*	*First Executive Order*
	*-0*.*358*	*-0*.323**
	*(0*.*257)*	*(0*.*156)*
ln(Population)	-5.982	-1.063
	(13.890)	(18.444)
ln(Elderly)	10.773	4.819
	(9.797)	(10.404)
ln(Income)	7.410**	7.819
	(3.070)	(5.169)
ln(Vehicle)	7.273***	8.181**
	(2.147)	(3.700)
ln(Commute)	0.466	0.025
	(0.396)	(0.684)
Temperature	0.037***	0.059***
	(0.002)	(0.008)
Rain	0.007	0.021
	(0.016)	(0.038)
Snow	-0.155***	-0.174***
	(0.032)	(0.037)
Wind	-0.025***	-0.029***
	(0.005)	(0.006)
Humidity	-0.014***	-0.025***
	(0.002)	(0.003)
Observations	4,161	1,736
R^2^	0.270	0.337
R^2^(Adjusted)	0.188	0.257
F-statistic	125.921***	71.657***
Daily fixed effects	Yes	Yes
City fixed effects	Yes	Yes

*** p<0.01

** p<0.05

* p<0.10

Robust standard errors are given in parentheses.

These results generally show the differences in residents’ travel behavior between weekdays, weekends, and bank holidays. After the first Covid-19 diagnosis, individuals might have started using bike-sharing platforms as an alternative to other modes of transportation on weekdays, especially, for journeys to and from work. However, with the first executive order implementation, on average, individuals might tend to stay inside more rather than go out. We also ran the analysis on daily trip frequencies with fewer than thirty minutes, as a single ride for non-subscribers includes thirty minutes of ride time. The results are consistent.

### Heterogeneity analyses

While our empirical estimations thus far suggest a significant impact of the Covid-19 pandemic on the frequency of bike-sharing platform trips, it is worth examining the factors that might amplify the strength of the effect. Prior literature [29–31] suggests that transportation infrastructure, land use, built environment, and neighborhood attributes contribute to individuals’ preference for bike-sharing systems.

One crucial factor that can moderate the impact of Covid-19 on bike-sharing platforms’ trip frequency is the pre-existing biking infrastructure. First, in cities with more bike lanes, longer bike route lengths, fewer hills, higher road connectivity, and bicycle-aware traffic, bike-sharing platforms should more likely be adopted by individuals and used as an alternative transportation mode. Second, in walkable cities with better access to amenities, residents might be embracing these platforms more due to easy and comfortable access to bike stations. Lastly, in cities with access to public transit, bike-sharing platforms might be used more by the residents due to better connectivity of the transit network. Therefore, we test the heterogeneous effects depending on such factors as the city’s bike-friendliness, transit-friendliness, and pedestrian-friendliness.

We collected data from Walk Score [40] to measure 1) bike-friendliness (*BikeScore*^®^) [41], which measures the built environment’s ability to support biking for a given location, 2) pedestrian-friendliness (*WalkScore*^®^) [41], which measures the walkability of any address by analyzing the walking routes to nearby amenities within a 5-minute walk, and 3) transit-friendliness (*TransitScore*^®^), which measures how well a location is served by public transit [41]. These measures range from 0 to 100 and divide cities into different groups [1]. Based on the classification of BikeScore^®^, WalkScore^®^, and TransitScore^®^, the cities in our dataset scored less than 90 in all measures. Detailed information on the groups and descriptive statistics of the scores are given in Tables 7 and 8, respectively.

**Table 7:** Description: BikeScore^®^, WalkScore^®^, and TransitScore^®^.

	BikeScore^®^	WalkScore^®^	TransitScore^®^
90–100	Biker’s Paradise	Walker’s Paradise	Rider’s Paradise
70–89	Very Bikeable	Very Walkable	Excellent Transit
50–69	Bikeable	Walkable	Good Transit
0–49	Somewhat Bikeable	Car-Dependent	Some/Minimal Transit

**Table 8:** Descriptive statistics: BikeScore^®^, WalkScore^®^, and TransitScore^®^.

Variable	Obs.	Mean	Std. Dev.	Min	Max
BikeScore^®^	6,052	67.71	9.84	49.90	83.50
WalkScore^®^	6,052	70.13	15.54	40.50	88.30
TransitScore^®^	6,052	61.43	16.33	32.80	84.30

Then, we re-estimate Eq (1) incorporating interaction terms for these classifications with the treatment. The new equation including the interaction terms is given below. Note that as the moderators are static, fixed-effects panel regressions do not yield estimates for *β*_2_. The results are given in Table 9.

**Table 9:** Bike-sharing trip frequency: Fixed-effects regression results by heterogeneous effects. The reference indicator for the bike-friendliness scale is Bikeable; the reference indicator for the pedestrian-friendliness scale is Walkable; and the reference indicator for the transit-friendliness is Good Transit.

	(1)	(2)	(3)	(4)	(5)	(6)
Dependent Variable	ln(TripFrequency)	ln(TripFrequency)	ln(TripFrequency)	ln(TripFrequency)	ln(TripFrequency)	ln(TripFrequency)
*Treatment variable*	*First Case*	*First Case*	*First Case*	*First Executive Order*	*First Executive Order*	*First Executive Order*
	*0*.314**	*0*.*049*	*0*.*078*	*-0*.189*	*-0*.429*	*-0*.387*
	*(0*.*152)*	*(0*.*194)*	*(0*.*113)*	*(0*.*112)*	*(0*.*025)*	*(0*.*213)*
Interaction: VeryBikeable	0.640***			0.708***		
	(0.228)			(0.225)		
Interaction: SomewhatBikeable	-0.405*			-0.356*		
	(0.215)			(0.194)		
Interaction: VeryWalkable		0.824***			0.833**	
		(0.312)			(0.324)	
Interaction: CarDependent		-0.020			-0.023	
		(0.186)			(0.198)	
Interaction: ExcellentTransit			0.797***			0.806***
			(0.263)			(0.272)
Interaction: SomeTransit			-0.106			-0.104
			(0.133)			(0.139)
ln(Population)	-28.236	-23.398	-13.360	-14.129	-14.108	-14.436
	(21.704)	(20.104)	(16.429)	(16.143)	(14.231)	(14.436)
ln(Elderly)	17.118	15.485	-5.067	-3.335	-4.122	-2.551
	(11.362)	(10.788)	(11.572)	(11.246)	(10.632)	(10.408)
ln(Income)	10.441**	9.591**	1.747	2.514	1.668	2.534
	(5.129)	(4.682)	(4.748)	(4.512)	(3.898)	(3.727)
ln(Vehicle)	2.032	2.517	2.087	2.545	2.322	2.659
	(2.710)	(2.677)	(3.753)	(3.484)	(3.314)	(3.139)
ln(Commute)	1.701***	1.647***	1.225**	1.182**	1.228**	1.219**
	(0.639)	(0.624)	(0.587)	(0.565)	(0.539)	(0.524)
Temperature	0.041***	0.041***	0.042***	0.041***	0.042***	0.042***
	(0.003)	(0.003)	(0.003)	(0.003)	(0.003)	(0.003)
Rain	0.004	0.010	0.006	0.008	0.008	0.011
	(0.024)	(0.021)	(0.024)	(0.022)	(0.023)	(0.020)
Snow	-0.173***	-0.174***	-0.173***	-0.173***	-0.173***	-0.173***
	(0.038)	(0.038)	(0.039)	(0.038)	(0.038)	(0.038)
Wind	-0.025***	-0.025***	-0.025***	-0.025***	-0.025***	-0.025***
	(0.005)	(0.006)	(0.005)	(0.005)	(0.005)	(0.004)
Humidity	-0.017***	-0.016***	-0.017***	-0.016***	-0.017***	-0.017***
	(0.002)	(0.002)	(0.002)	(0.002)	(0.002)	(0.002)
Observations	6,052	6,052	6,052	6,052	6,052	6,052
R^2^	0.326	0.323	0.325	0.330	0.326	0.328
R^2^(Adjusted)	0.252	0.248	0.250	0.257	0.252	0.254
F-statistic	203.65***	200.193***	201.941***	207.319***	203.57***	205.077***
Daily fixed effects	Yes	Yes	Yes	Yes	Yes	Yes
City fixed effects	Yes	Yes	Yes	Yes	Yes	Yes

*** p<0.01

** p<0.05

* p<0.10

Robust standard errors are given in parentheses.


$$\text{ln}{\left(TripFrequency\right)}_{ij}=\;\beta_{0}+\beta_{1}Treatment_{ij}+\beta_{2}Moderator_{j}+\beta_{3}Treatment_{ij}*Moderator_{j}+\gamma W_{ij}+\;\theta_{j}+\;\mu_{i}\;+\varepsilon_{ij},$$ (2)

Surprisingly, these findings suggest interesting differences. First, we see that the impact of the first Covid-19 case and the first executive order implementation on bike-sharing platforms’ trip frequency is more substantial in bikeable cities. We estimate that the effect of the first Covid-19 case on Trip Frequency is approximately 90% (rounded from the following: [exp(0.640)-1]*100 = 89.64%) higher in “*very bikeable*” cities than in “*bikeable*” cities (*see* Table 9, *Column 1*). We observe that the impact of the first executive order implementation on Trip Frequency is approximately 103% (rounded from the following: [exp(0.708)-1]*100 = 102.99%) higher in “*very bikeable*” cities than in “*bikeable*” cities (*see* Table 9, *Column 4*). Followed by the first Covid-19 case and the first executive order implementation, we estimate a decrease that is respectively greater by approximately 33% (rounded from the following: [exp(-0.405)-1]*100 = -33.30%) (*see* Table 9, *Column 1*) and 30% (rounded from the following: [exp(-0.356)-1]*100 = -29.95%) (*see* Table 9, *Column 4*) in Trip Frequency in “*somewhat bikeable*” as compared to “*bikeable*” cities. These results might suggest that the residents in bike-friendly cities embrace these platforms more due to better pre-existing biking infrastructure. With safe and comfortable biking afforded by good biking infrastructure, residents are more likely to use bike-sharing platforms for commuting and recreational purposes.

Moreover, we observe a more substantial impact on the Trip Frequency in very walkable cities upon both the first Covid-19 case (*see* Table 9, *Column 2*) and stay-at-home advisory implementation (*see* Table 9, *Column 5*). In pedestrian-friendly cities, residents might be embracing these platforms more as a result of easy and comfortable access to bike stations by walking. Moreover, similar to bike-friendliness and pedestrian-friendliness, we observe a more substantial impact on the Trip Frequency in cities with excellent transit upon both the first Covid-19 case (*see* Table 9, *Column 3*) and the first executive order implementation (*see* Table 9, Column 6). Lastly, unlike the “*somewhat bikeable”* classification of cities, we do not observe that car dependence or having *some transit* (compared to *good* transit) has any moderating influence on the effect of the first Covid-19 case (*see* Table 9, *Column 3*) and the first executive order implementation (*see Table* 9, *Column 6*) on the use of bike-sharing platforms.

## Discussion and conclusion

We used a DID framework formulated as a fixed-effects regression model to examine how bike-sharing trip frequency in the United States changed with the onset of the Covid-19 pandemic. We modeled the first reported Covid-19 cases and the implementation of the first executive order in each municipality as two treatment events. We also accounted for socio-economic factors, weather, and fixed effects for each day and city. First, our results indicate that, on average, the first reported Covid-19 cases had a *positive* and statistically significant effect on the frequency of bike trips in U.S. cities. This could be explained by the fact that the existence of the first reported Covid-19 case in U.S. cities has heightened individuals’ sensitivity to cleanliness and social distance. Therefore, individuals were compelled to change their travel behavior and look for alternative systems of mobility that may offer more resilient urban transportation. Bike-sharing platforms offer alternative transportation to avoid crowds in the cities. Second, we observe that the first executive order advisories had a *negative* and statistically significant effect on the frequency of bike trips in U.S. cities. This could be explained by the fact that lockdown restrictions and working from home have led to a decline in commuting bike trips and other modes of transportation such as public transit.

We also examined sources of heterogeneity in the effect of the pandemic on bike-sharing use. We compared how bike-sharing use changed between weekends and weekdays with the onset of the pandemic. We observed an increase in weekday-specific trip frequency as a result of the first Covid-19 case diagnosis and a decrease in weekend-specific trip frequency due to the first executive order implementation. We also tested for heterogeneous impacts across a set of city-level characteristics. We found that there is a greater increase in the frequency of bike-sharing trips in more bike-friendly, transit-friendly, and pedestrian-friendly cities upon both the first Covid-19 case diagnosis and the first executive order implementation. We might conclude that bike-sharing platforms have an essential role in individuals’ travel behavior, especially in cities with better bike and transit infrastructure. These platforms are perceived as a sustainable and resilient transportation option by individuals due to the unprecedented consequences of the Covid-19 pandemic.

Bike-sharing platforms offer a sustainable and active mode of transportation, and hence it is important to better understand the factors that affect their adoption by the populace. The Covid-19 pandemic represents an opportunity for cities to embrace new paradigms for urban mobility. Bike-sharing platforms represent one way in which cities provide a resilient and adaptive transport network to face the potential challenges of disruptive events like the Covid-19 pandemic. The pandemic has already highlighted the importance of rethinking the design of urban transit for greater resilience to such disruptive events. Cities may consider how to encourage greater use of bike-sharing platforms. Decisions by city authorities such as offering free or reduced membership could break down barriers to the adoption of bike-sharing. With the support of local authorities in creating more bike lanes and accessibility to public spaces, bike-sharing platforms can attract more individuals. Proper incentives followed by infrastructure adjustments could ensure that individuals will become accustomed to bike-sharing platforms and continue to use them even after the pandemic. For instance, in New York, city officials are already planning to expand Citi Bike and add more docking stations in its busiest areas [12]. Investing in bike-sharing platforms and cycling infrastructure could lead to an increase in memberships because individuals’ willingness to bike is closely linked to how safe they feel [42].

It is important to note that our research is subject to several limitations. First, the adjusted R^2^ values of the models are low, ranging from 0.18 to 0.26. Low values might be an indicator of the models would not be suitable for use in the predictive modeling of the outcome variables. Hence, the aims of our model interpretations are limited to assessing the direction and significance of coefficient estimates for causal inference.

Second, the main challenge with DID estimation is to ensure that no pre-treatment trends in the absence of treatment [25]. The violation of this assumption can lead to biased causal estimates. Although there is no statistical test for this assumption, we examine the robustness of our model to temporal trends using a relative time model. Our findings suggest that there are no pre-existing trends in bike-sharing demand across the cities that experience the first Covid-19 diagnosis (*see Fig 6A in* S1 Appendix). As seen in Fig 7A in S1 Appendix, we observe that pre-treatment trends exist in bike-sharing demand across the cities experiencing the first executive order implementation. Future research could explore different estimators that could overcome this challenge. There are a few recent papers that focus on different ways to relax this assumption [43–45]. They propose alternative estimators when the parallel trends assumption is violated and examine the robustness of the results to the potential violations of parallel trends.

Third, our model only examines the short-term effect of the Covid-19 pandemic on bike-sharing demand due to the data collection period. In the future, depending on data availability by the bike-sharing providers, the effects can be examined for longer periods.

Fourth, we use the first executive order implementation and the first Covid-19 case as proxies of the Covid-19 pandemic; however, we should keep in mind that the first executive order implementation might be correlated with the first Covid-19 case. This might be also one of the reasons that might explain the pre-treatment trend in Fig 7A in S1 Appendix.

Lastly, our reported results are based upon the actual usage of bike-sharing platforms, along with public transit data for eleven cities in the U.S. Despite the lack of data available for other U.S. cities, we believe that the exogenous nature of the Covid-19 pandemic provides robust insights into the relationship between the Covid-19 pandemic and travel behaviors. Given that we are still in the midst of the pandemic, we expect that forthcoming data could reveal more about the pandemic’s long-term effects on travel behavior. It is very plausible that the effects observed thus far may serve as a signal for more lasting changes to come in urban travel behaviors.

## Supporting information

S1 Dataset (CSV)

S1 Appendix (DOCX)
