# Supplementary material for: When crisis hits: Bike-Sharing platforms amid the Covid-19 pandemic
Source: PLoS One. 2023 Apr 7;18(4):e0283603. doi: 10.1371/journal.pone.0283603 (PMC10081752; doi:10.1371/journal.pone.0283603)
Supplement: S1 Appendix — (DOCX) [file pone.0283603.s002.docx]

**Appendix**

**Model Free Evidence**

Similar to the plots in Fig 1 and Fig 2 in the manuscript, we plot the difference in the bike-sharing trip frequency before and after the first Covid-19 case diagnosis and the first executive order implementation by weekday and weekend. In Fig 1A, we show the weekday bike trip frequencies 60 days before and after the first Covid-19 case reported in each city, whereas in Fig 2A, we show the weekend bike trip frequencies 60 days before and after the first Covid-19 case that was reported.


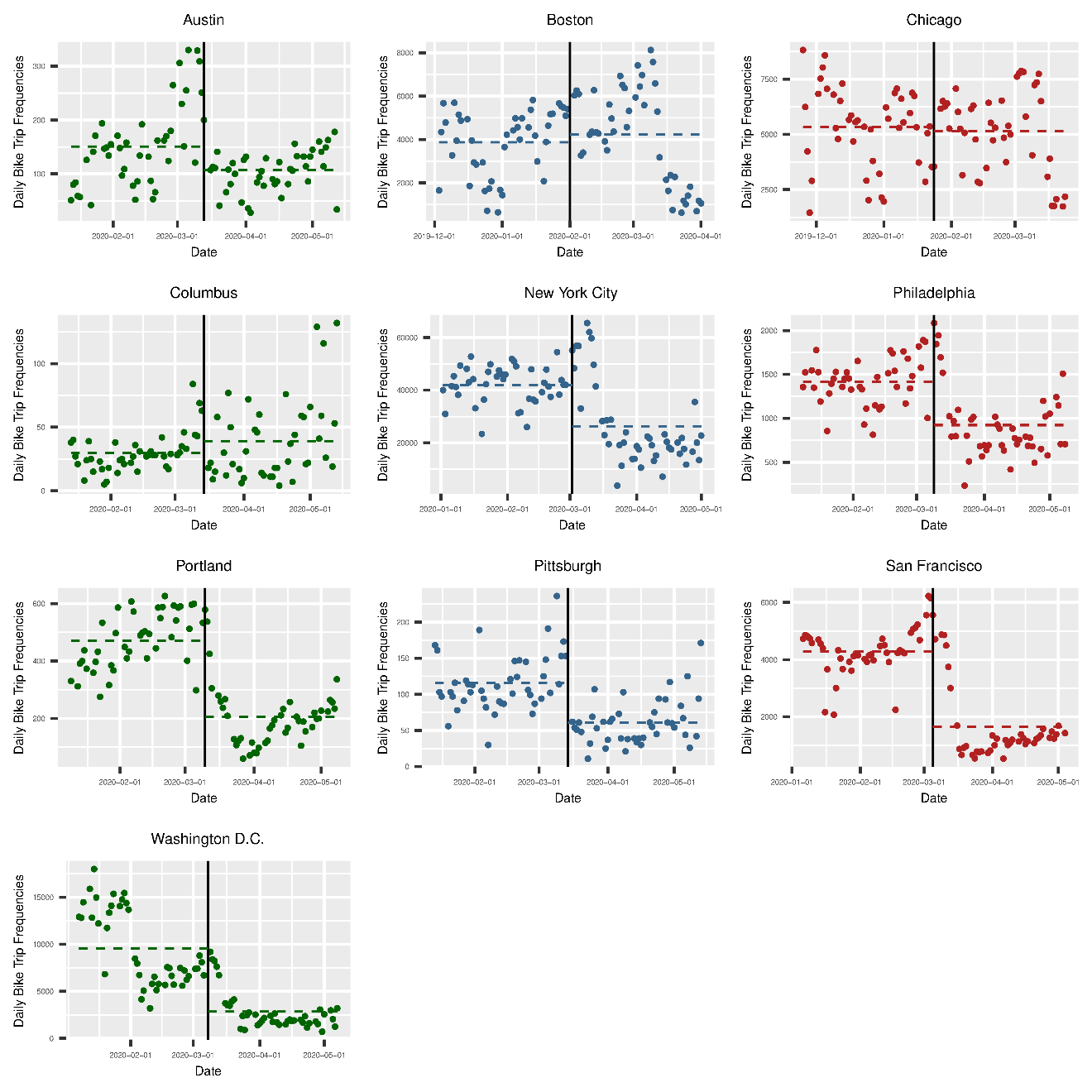


**Fig 1A**. Frequency of weekday bike-sharing platform trips over time, before and after the first Covid-19 case.


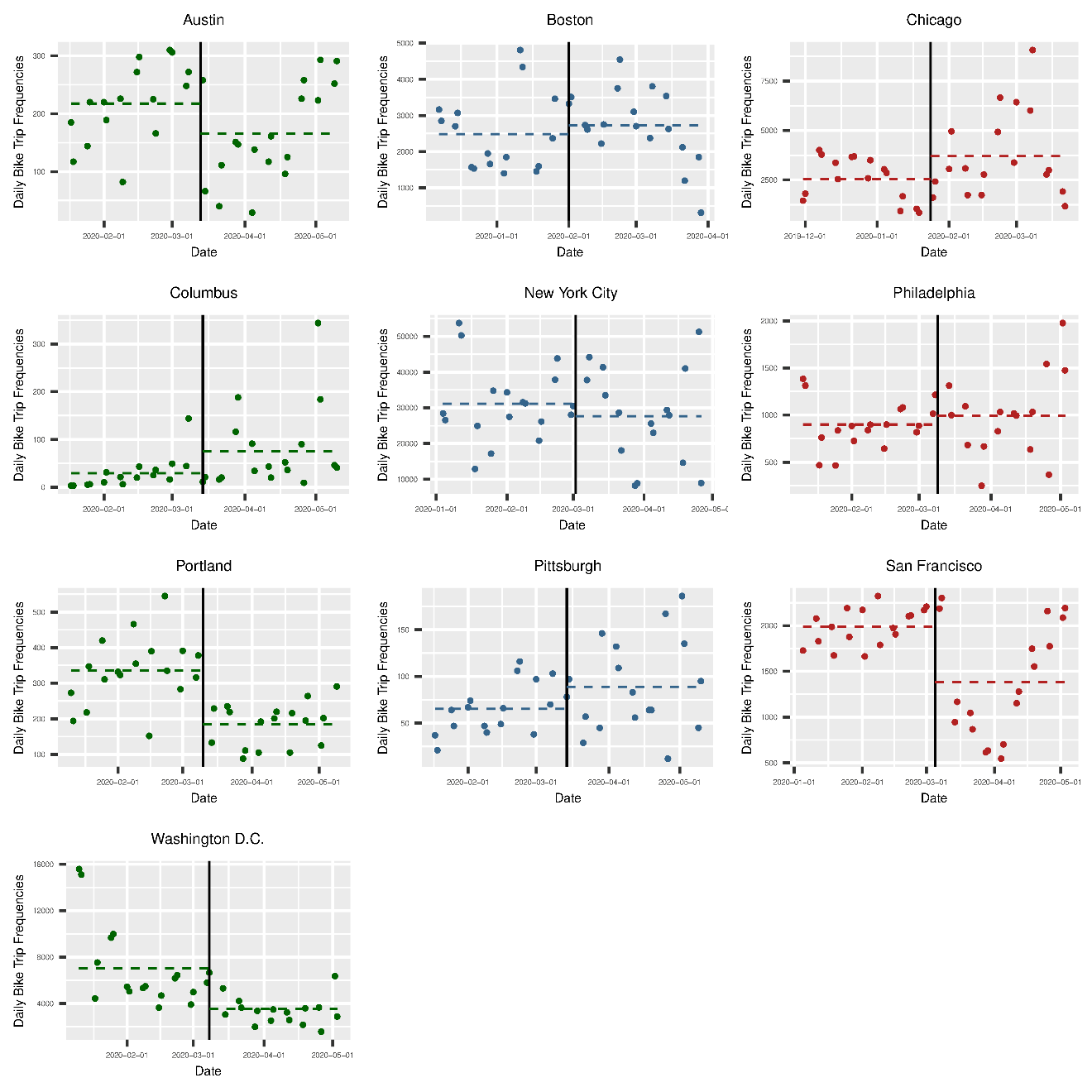


**Fig 2A**. Frequency of weekend bike-sharing platform trips over time, before and after the first Covid-19 case.

Overall, we notice a decrease in the trip frequency following the first reported Covid-19 case in most cities, with few exceptions in which we see a close average daily trip frequency after the first Covid-19 case was reported, such as in Boston. We observe a similar pattern in Figs 3A and 4A, which present the weekday and weekend trip frequencies before and after the first executive order implementation in each city, respectively. However, Columbus and Pittsburgh experience an increase in the weekend trip frequencies following the first executive order. This could be due to factors such as weather changes.


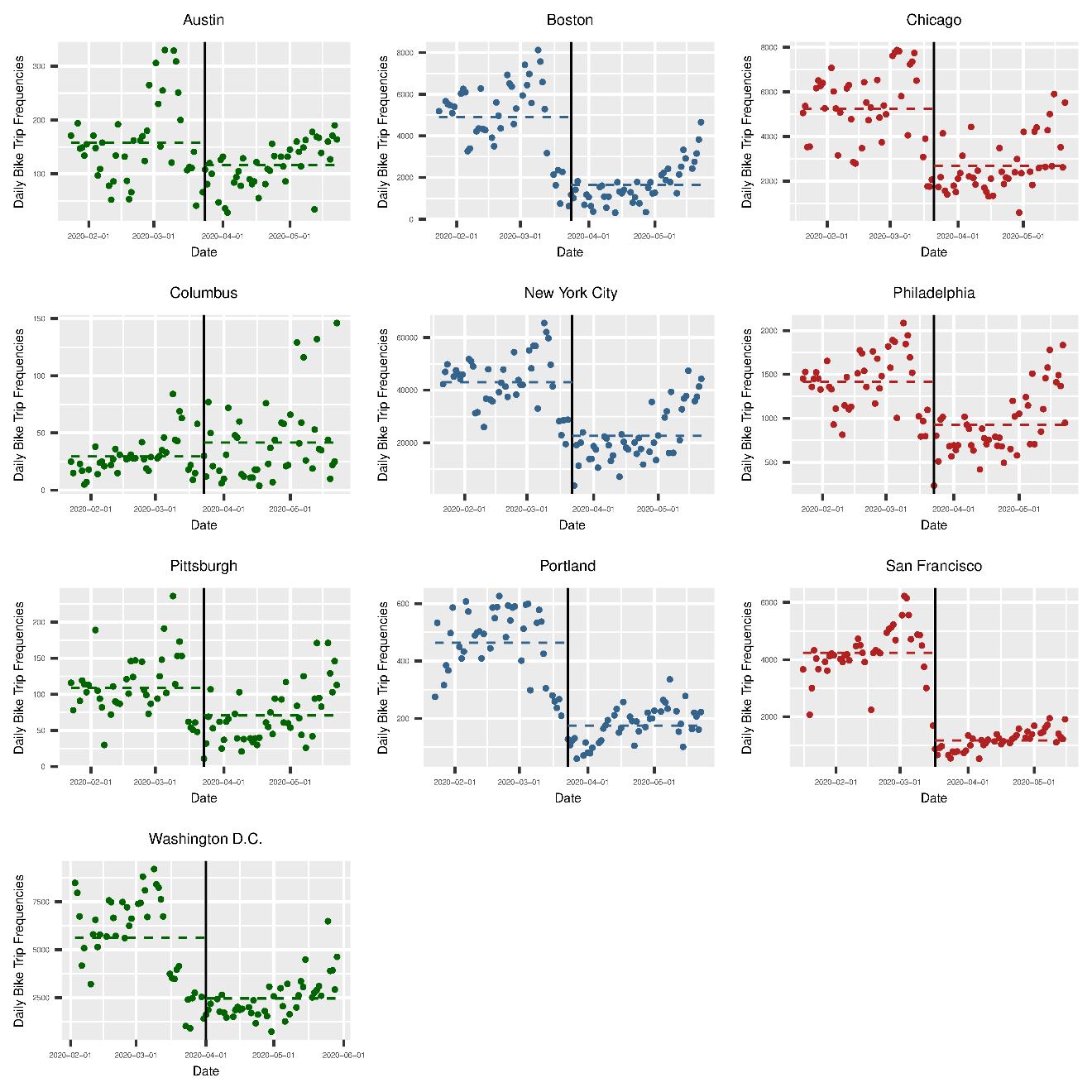


**Fig 3A**. Frequency of weekday bike-sharing platform trips over time, before and after stay-at-home advisory implementation.


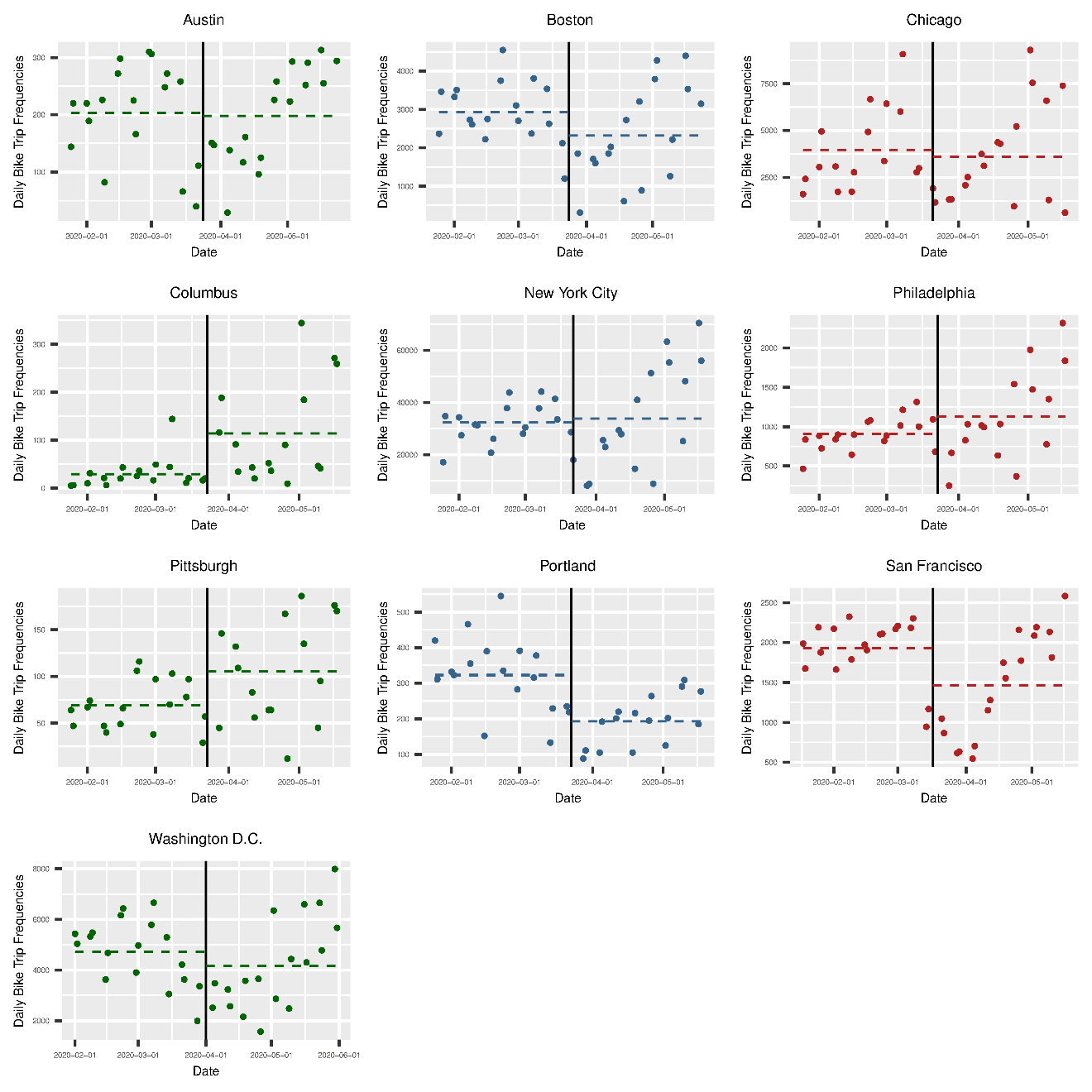


**Fig 4A**. Frequency of weekend bike-sharing platform trips over time, before and after the first executive order implementation.

Fig 5A shows the bike-sharing seasonal trend of February-June 2019 (pre-covid) compared to February-June 2020 (post-covid) for each city in this study. Relative to 2019, cities experienced a short-term decrease in bike-sharing trip frequency following the start of the pandemic (towards the end of the first quarter of 2020). These plots provide further model-free evidence of the changes in the use of the bike-sharing system due to Covid-19.


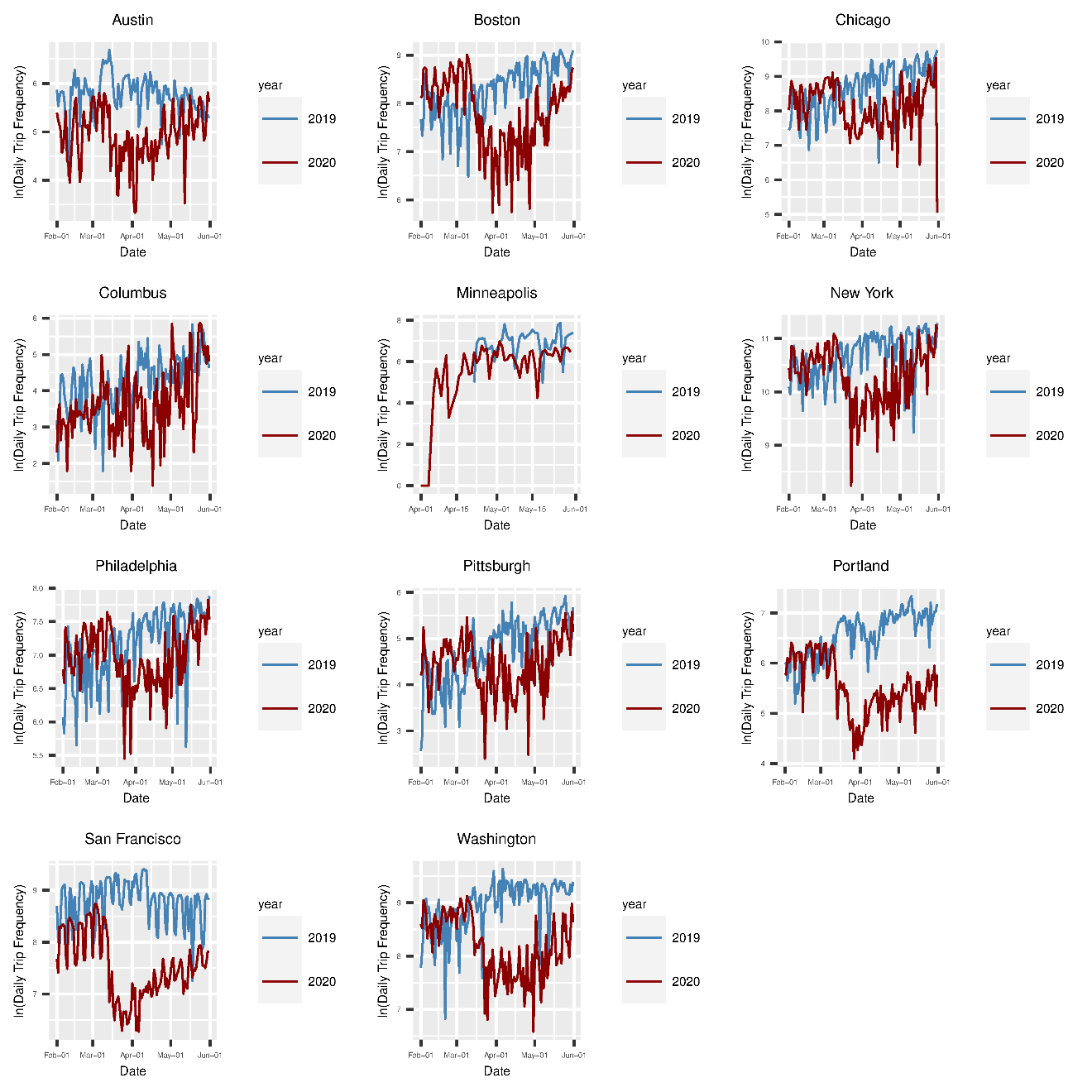


**Fig 5A.** Bike-sharing trip frequency trend 2019 vs. 2020

**Relative Time Model**

One of the critical assumptions of the DID framework is that pre-treatment trends of the outcome should be the same between treated and untreated units prior to the treatment. [1] Violation of this assumption leads to a biased estimation of causal effects. Although there is no statistical test for this assumption, we examine the robustness of our model to temporal trends using a relative time model. We set time dummies representing the relative temporal distance, *k*, between an observation period, *t*, and the timing of bike-sharing platform entry in the city *i*. The relative time model is specified as follows:

$ln(TripFrequency)ij= \sum_{k} \beta_{k}{Treatment}_{ij}\left( k \right)+ \gamma W_{ij}+ \theta_{j}+ \mu_{i} +\varepsilon_{ij}$, (A1)

where *ln(TripFrequency)_ij_* is the log-transformed value of our dependent variable in city *i* during day *j*; $\mu_{i}$ is the city fixed effects; $W_{ij}$ is the set of control variables; and *Treatment_ij_(k)* is the vector of relative time dummies, which is set to one of the relative temporal distance between treatment entry’s into city *i* in day *j* is *k*. Our empirical estimates for Equation (A1) are given in Figs 6A-7A. Fig 6A suggests that we do not observe statistical significance in the pretreatment time dummies, indicating that there are no pre-existing trends in bike-sharing demand across the cities that experience the first Covid-19 diagnosis. We also observe that the trend goes up after the treatment, which is consistent with our main results. On the other hand, we observe significant pre-existing trends in bike-sharing demand across the cities that experience the first-executive order implementation (*see Fig 7A*). However, rather than abandoning this treatment, future research could explore different estimators that could overcome this challenge. A few recent papers focus on different ways to relax this assumption [2-4]. They propose alternative estimators when the parallel trends assumption is violated and examine the robustness of the results to the potential violations of parallel trends.


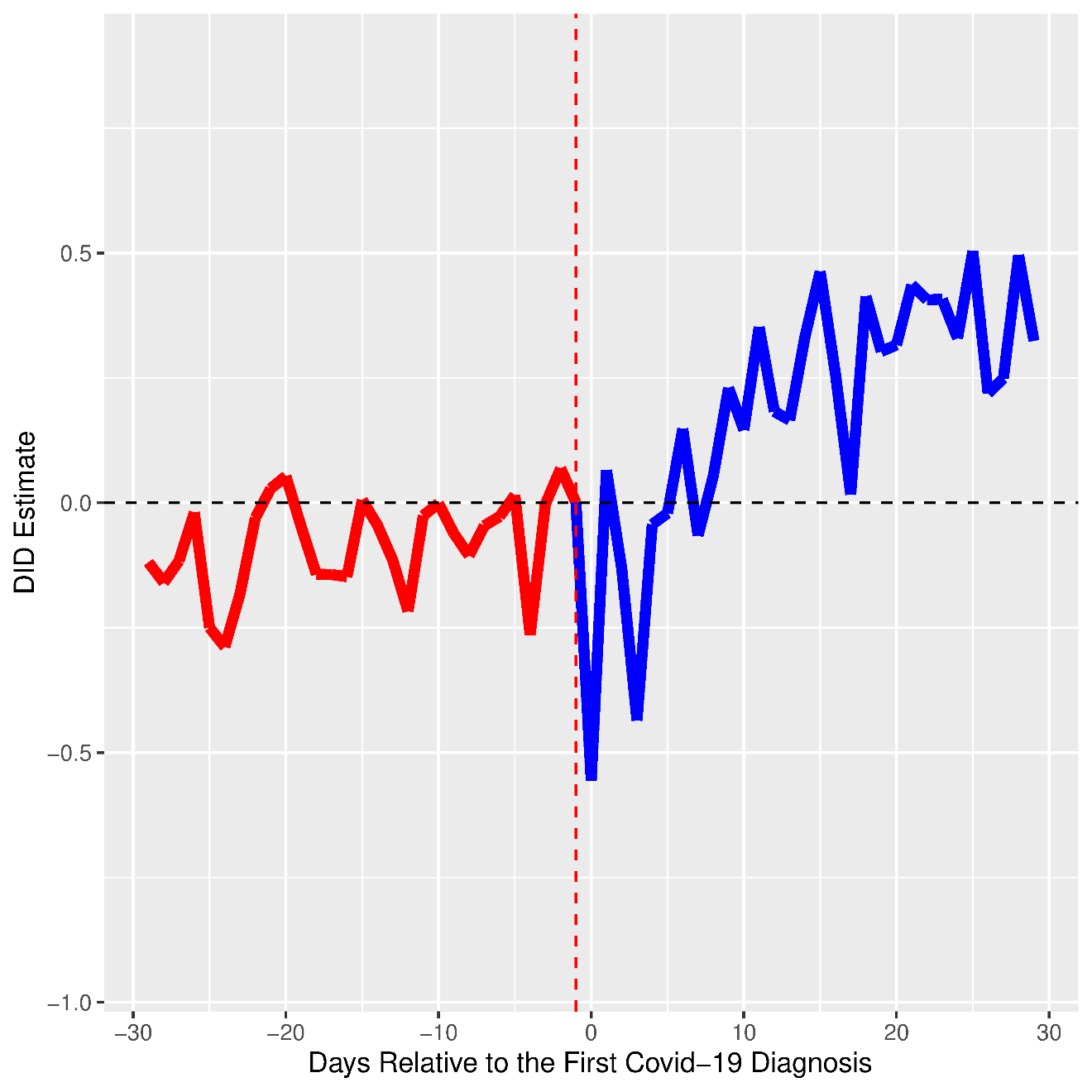


**Fig 6A**. Relative time model analysis results where the treatment is the first Covid-19 case


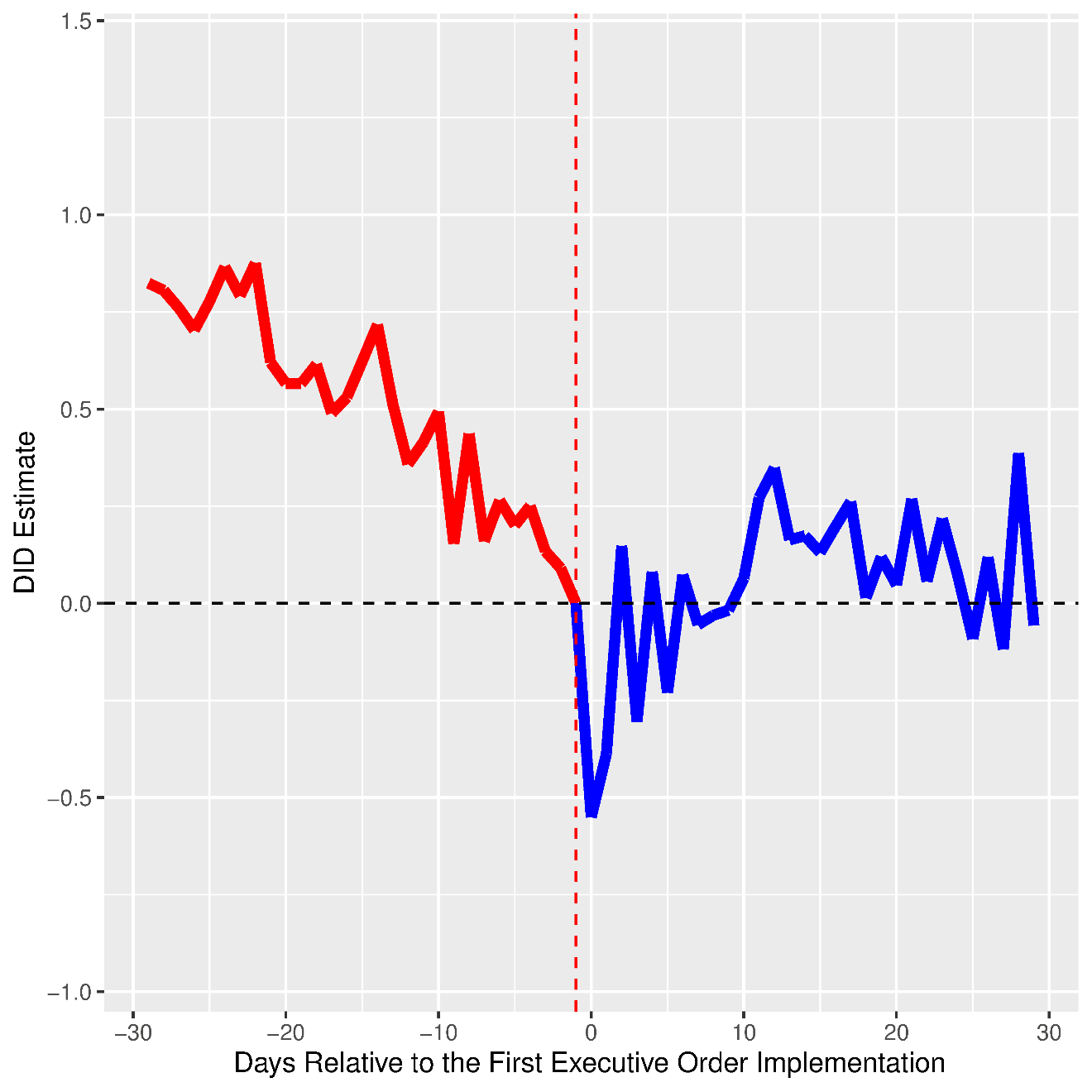


**Fig 7A**. Relative time model analysis results where the treatment is the first executive order implementation

**References**

1. Angrist JD, Pischke J-S. Mostly harmless econometrics: An empiricist’s companion. Princeton University Press. 2008. doi:10.1017/CBO9781107415324.004

2. Rambahan A, Roth C. A More Credible Approach to Parallel Trends. 2022; Working paper.

3. Bilinski A, Hatfield, LA. Nothing to see here? Non-inferiority approaches to parallel trends and other model assumptions. 2018; arXiv preprint arXiv:1805.03273

4. Freyaldenhoven S, Hansen C, Shapiro JM. Pre-event trends in the panel event-study design. American Economic Review. 2019; 109: 3307-8.
